# The Enticing Path of miR Therapeutics: Difficult but Not without Prospects

**DOI:** 10.3390/cells11030418

**Published:** 2022-01-26

**Authors:** Ajit Vikram

**Affiliations:** Department of Internal Medicine, Carver College of Medicine, The University of Iowa, Iowa City, IA 522442, USA; ajit-vikram@uiowa.edu

## 1. Introduction

MicroRNAs (miRs) are short non-coding RNAs that regulate the translation and stability of mRNAs to fine-tune gene expression. Over 2000 miRs in humans control tens of thousands of genes, a single miR targets hundreds of genes, and the levels of miRs are deregulated during disease pathogenesis and recovery. The presence of more miRs in complex animals and their dysregulation during illness corroborate their biological importance. The ability of miRs to target multiple genes, including those that encode for traditionally undruggable proteins, demonstrates their suitability as a target. In this collection, we present the current developments in the role of miRs (e.g., miR-511, miR-27-3p, and miR-214) in disease conditions (e.g., stroke, cancer, androgenic alopecia), their suitability as biomarkers, and challenges in producing miR therapeutics.

## 2. miRs in Health and Disease

### 2.1. miR-214

This is known to play a role in multiple disease conditions (e.g., gastric cancer, lung cancer, heart failure). This collection includes an up-to-date summary of findings on the role of miR-214 in health and disease [1].

### 2.2. miR-27a

The pre-miRs generate two strands of miRs (3p and 5p), and strand sorting depends on multiple factors. Both strands can target a distinct set of genes, and in many cases, one strand is rapidly degraded. Both strands of miR-27a (miR-27a-3p and miR-27a-5p) are downregulated during adipogenic differentiation. Though, which strand contributes to the adipogenesis remained unknown. Wu et al. show that the miR-27a-3p, but not miR-27a-5p, contributes to adipogenic differentiation by targeting PPARγ [2].

### 2.3. miR-141-3p

Ischemic stroke is a vascular disorder where the blood supply to a part of the brain is obstructed, resulting in injury in that part of the brain. In such conditions, studies show a change in the levels of miRs, including miR-141-3p. miR-141-3p inhibition alleviates the negative impact of post-stroke social isolation in aged mice [3]. However, the delivery of miRs to the brain and their non-specific accumulation in different tissues has been a challenge. In this issue, Dhuri et al. compared the therapeutic efficacy of the nanoparticle-delivered miR-141-3p inhibitor synthesized by either peptide nucleic acid or phosphorothioates technology. They found that the former has superior efficacy at rescuing ischemic stroke injury [4]. This study shows the suitability of PNA technology as miR inhibitors and demonstrates the usefulness of nanotechnology for miR inhibitor delivery.

### 2.4. miR-18a

Long non-coding RNAs (lncRNA) have single or multiple binding sites for miRs and compete with the target genes to bind with the miR. López-Camarillo et al. reviewed the current knowledge on lncRNA and miR interplay in mRNA regulation in the context of ovarian cancer. They described some interesting connections based on the published information (e.g., lncRNA WDFY3-AS2: miR-18a interaction regulates RORA expression, which plays a crucial role in ovarian cancer [5,6]).

### 2.5. miR-511

This promotes intestinal inflammation by regulating the TLR4 responses, but the mechanism remains unknown. Rahman et al. show that the inflammatory effect of miR-511 is mediated by regulation of TLR3/4 response via wdfy1 [7].

### 2.6. hsa-miR-30a-3p and hsa-miR-139-5p

The discussion on miR’s role in disease conditions seems incomplete without talking about COVID-19. In this issue, Li et al. show that the hsa-miR-30a-3p and hsa-miR-139-5p have a causal effect on the development of severe COVID-19.

### 2.7. Group of miRs

SIRT2, a sirtuin family member, is abundantly expressed in neuronal cells’ cytosol. The single nucleotide polymorphisms (e.g., rs2241703, rs2015) in the miR binding sites of SIRT2 3′-UTR have been identified in the patients with neurodegenerative diseases. The review by Kaitsuka et al. highlights the SIRT2 regulation by miRs in the context of aging-related conditions [8]. The DKK1 and Wnt/β-catenin regulation are critical approaches in promoting hair growth. Papukashvili et al. reviewed the miRs (e.g., miR-103/107, miR-203, and miR-218) that could modulate the Wnt/β-catenin signaling pathway via regulating DKK1 and represent attractive therapeutic candidates [9]. Recent studies have shown the role of miRs in the pathogenesis of inflammatory bowel disease. Stiegler et al. focus on the miRs that directly and indirectly target genes associated with gut permeability regulation [10].

## 3. miRs as Biomarkers

The study by Martinez-Fierro et al. compared the serum miR profile at different stages of gestation and at the time of preeclampsia (a condition of high blood pressure during pregnancy). They found that select miRs (e.g., miR-628-3p, miR-151a-3p, and miR-573) are differently expressed in the serum of women before they developed preeclampsia and suggested their predictor and contributor potential [11]. Ebstein’s Anomaly is a congenital cardiovascular disorder where the heart’s tricuspid valve is not formed correctly, leading to regurgitation of blood and decreased cardiac efficiency. In this issue, Abu-Halima et al. compared the levels of miRs and mRNAs in the blood of Ebstein’s Anomaly patients and age/sex-matched healthy controls. They employed high throughput analysis of miRs and mRNAs interactions using bioinformatics tools to identify the deregulated signaling pathways (e.g., 5HT2 receptor, alpha-adrenergic receptor, and Alzheimer’s disease) [12]. Kennel et al. reviewed the miRs (e.g., miR-499, miR-208, miR-1) that can be used as biomarkers for diagnosing heart failure. The primary concerns are the low reproducibility of miR-based biomarkers and the absence of added advantage over protein-based assays [13].

## 4. miR Therapeutics: Opportunities and Challenges

miRs have been implicated in almost every facet of molecular networks. However, many animals endure harsh environmental conditions in nature (e.g., cold/freezing temperatures, oxygen limitation, food or water scarcity), which requires them to revamp their metabolic organization. In an exciting and well-written review, Singh et al. describe the role of miRs in such adaptive processes with putative applications (e.g., organ preservation, inflammation, aging, metabolic disorders, mitochondrial dysfunction). The species-specific comparison and conserved miR responses in evolutionarily disparate animal species can help us understand the complex miR network regulating metabolism to achieve diverse outcomes [14]. Despite several studies revealing the therapeutic potential of miRs, just 20 miR-therapeutics have entered clinical trials, with none progressing to phase III trials. A lower number of miR therapeutics in the pipeline suggests an unexplainable hurdle in developing miR therapeutics [15]. In this issue, Momin et al. summarized the challenges and opportunities in developing miR therapeutics from a multidisciplinary view [16]. In conclusion, the journey to developing miR therapeutics and identifying it as a biomarker is attractive but full of hurdles because of its ubiquitous expression, non-specificity, and delivery challenges. However, recognizing the biologically significant miRs in diseases at higher resolution, developing the miR-inhibitors with higher specificity to the target and lower non-specific interactions, and miRs derived from plants are some of the emerging opportunities that hold promise for miR therapeutics. I hope that this collection will provide a balanced update about the current developments in the role of miRs in health and disease, opportunities, and challenges through research and review articles.

## Data Availability

Not applicable.
